# Adapting existing toxicokinetic models to relate perfluoroalkyl and polyfluoroalkyl intake to biomarkers in humans

**DOI:** 10.1093/toxsci/kfaf087

**Published:** 2025-06-16

**Authors:** Kara J Dean, Régis Pouillot, Jane M Van Doren, Sofia M Santillana Farakos

**Affiliations:** Human Foods Program, U.S. Food and Drug Administration, College Park, MD 20740, United States; Consultant, Rabat 10170, Morocco; Human Foods Program, U.S. Food and Drug Administration, College Park, MD 20740, United States; Human Foods Program, U.S. Food and Drug Administration, College Park, MD 20740, United States

**Keywords:** compartmental model, toxicokinetics, animals, perfluorinated chemicals, biomarkers, mathematical modeling

## Abstract

Exposures to per- and polyfluoroalkyl substances (PFAS) are associated with various adverse health outcomes, and a wide range of PFAS compounds have been detected in human serum, the environment, and food. Toxicokinetic models, however, have been developed for only a subset of the compounds of interest. To facilitate reverse dosimetry and risk assessment for the less studied PFAS compounds in food, we developed and evaluated an approach to adapt existing toxicokinetic models for nonhuman primates to predict human serum levels. The approach was validated with perfluorooctanoic acid and perfluorooctanesulfonic acid data and applied to perfluorohexanesulfonate. Results indicate that the approach yields similar dosimetry estimates to those of other models, particularly those used for regulatory purposes, suggesting the methodology can be leveraged to inform decision-making in data-sparse spaces. Applying and adapting the framework will improve our ability to connect dietary PFAS exposures to endpoints of concern for a wide range of PFAS compounds.

Per- and polyfluoroalkyl substances (PFAS) are a class of synthetic chemicals composed of thousands of compounds that are known to be highly persistent in the environment. Available epidemiological studies indicate associations between exposure to perfluorooctanoic acid (PFOA) and perfluorooctanesulfonic acid (PFOS) and several health outcomes, including decreased antibody response, increases in serum lipids, pregnancy-induced hypertension, increases in serum hepatic enzymes, and decreased birth weights (ATSDR 2021; US EPA 2024a, 2024b). Exposure to other PFAS, such as perfluorononanoic acid (PFNA), perflourodecanoic acid (PFDA), perfluorohexanesulfonate (PFHxS), perfluorobutanoic acid (PFBA), and perfluorobutanesulfonic acid (PFBS), has also been reported to be associated with developmental, thyroid, immune, hepatic, kidney, and/or reproductive effects (Gleason et al. 2015; ATSDR 2021; US EPA 2021, 2022, 2023).

Higher concentrations of PFAS in serum have been detected in some individuals with occupational exposures and some residents of communities with proximity to military sites, commercial airports, landfills, or fluorochemical manufacturers (Calafat et al. 2019; ATSDR 2021). However, for most individuals, the primary routes of exposure are considered to be the ingestion of contaminated water and food (Domingo and Nadal 2017). A study by the US Centers for Disease Control and Prevention reports serum to be the best biomarker for biomonitoring PFAS, regardless of biopersistence, and summarizes National Health and Nutrition Examination Survey (NHANES) biomonitoring data from 2013 to 2014 in serum indicating universal exposure to PFOS, PFOA, PFNA, and PFHxS among the US general population (Calafat et al. 2019).

Toxicokinetic (TK) modeling is used to describe and quantify the relationship between external measures of exposure (i.e. food intake) and internal biomarkers (i.e. serum). Compartmental models are considered the simplest form of TK model in which a system is represented by one or few compartments, and modified compartment models have extensions that can also describe the toxicokinetics during pregnancy and lactation. Physiologically based toxicokinetic (PBTK) models are the most complex TK models, in which the concentration of a substance is modeled in multiple tissues synchronously using physiologically based parameters. PBTK models are the most physiologically accurate, but the model complexity is data-intensive.

Detailed reviews of available PFAS TK models have been published elsewhere (Chou and Lin 2021; East et al. 2023; US EPA 2024a, 2024b). Briefly, several TK models, including animal, human, PBTK, as well as compartmental models, have been published for the long-chain PFOA and PFOS compounds (e.g. Andersen et al. 2006; Tan et al. 2008; Wambaugh et al. 2008; Wambaugh et al. 2013; Loccisano et al. 2011; Verner et al. 2016; Ruark et al. 2017; Brochot et al. 2019; Chou and Lin 2019; Goeden et al. 2019). A few of these compartment and PBTK models have been applied for regulatory purposes (Loccisano et al. 2011; Wambaugh et al. 2013; Verner et al. 2016; Goeden et al. 2019). There are fewer TK models (e.g. Fabrega et al. 2015; Kim et al. 2018, 2019; Sweeney 2022) available, however, for the large number of additional compounds that can be found in foods (e.g. PFDA, PFHxS, PFNA, PFBS), and the implementation of compartment developmental and PBTK models for compounds such as PFHxS, PFBS, and PFNA has shown inconsistencies with empirical data, which has limited their utility for risk assessments (Fabrega et al. 2015; Verner et al. 2016; Kim et al. 2018; Sweeney 2022; US EPA 2023, 2024c).

To facilitate TK modeling for compounds with limited human data, this study aims to develop an approach that makes use of existing animal data and models and scales it to humans. To achieve this goal, we developed an approach that adapts an existing TK model for nonhuman primates and relates PFAS exposures to the concentration of PFAS in the serum for humans. Given the available data for PFOS and PFOA, the approach was developed and validated for those 2 compounds and was then used to parameterize models for PFHxS forward and reverse dosimetry. The framework presented herein can be used to parametrize models for additional compounds, as data permits, and will improve the ability to better relate dietary PFAS exposures to endpoints of concern for a wider range of PFAS compounds.

## Materials and methods

### Selected model

Given the high number of compounds of interest found in food that are associated with minimal human-specific data for calibration, we selected the animal model in Wambaugh et al. (2013) for further adaptation herein. Importantly, the multi-compartment animal model in Wambaugh et al. (2013), referred to hereafter as the Wambaugh model, can accommodate nonlinear elimination patterns, which will maximize the utility of the TK modeling approach for leveraging animal datasets and will facilitate applications of the models for normal and extreme exposure scenarios.

**Fig. 1.**
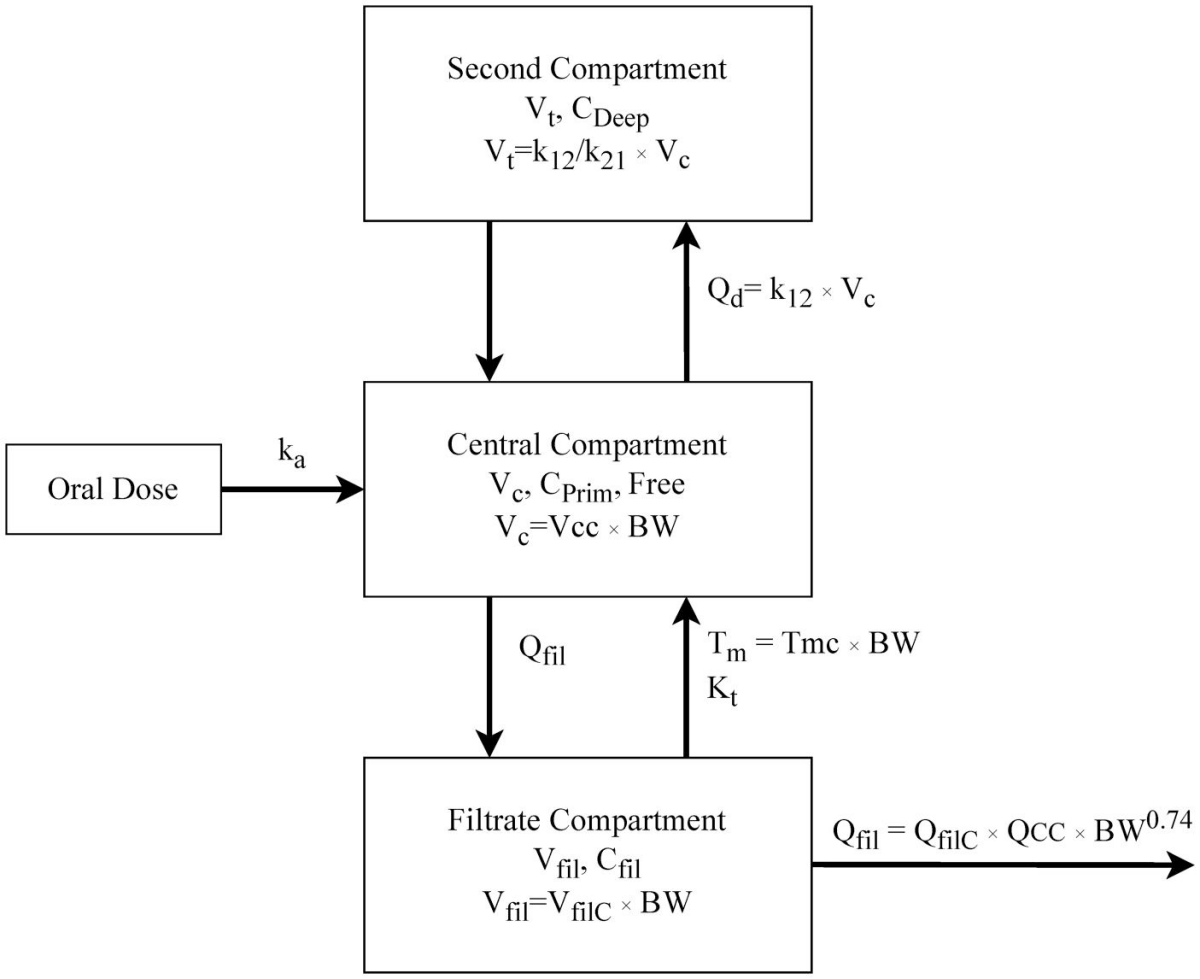
Schematic for the three-compartment model (*V_c_*, *V_t_*, *V_fil_*) with a saturable resorption process (*T_m_*, *K_t_*) adapted from Wambaugh et al. (2013) and Andersen et al. (2006); *C_Prim_*, *C_Deep_*, and *C_fil_* are the chemical concentrations in the primary/central, deep/second, and filtrate compartments.

The Wambaugh model is an adaptation of the Andersen et al. (2006) model and includes three compartments: A central compartment, a second (“deep”) compartment, and a filtrate compartment, where a saturable process with a Michaelis–Menten kinetics dictates reabsorption from the filtrate compartment to the central compartment (Fig. 1) (Andersen et al. 2006).

### Experimental data on PFOS and PFOA

In Wambaugh et al. (2013), the PFOS and PFOA pharmacokinetic models were fitted to cynomolgus monkey data originating from Butenhoff et al. (2004), Chang et al. (2012), and Seacat et al. (2002). We obtained the raw data for the PFOS experiments directly from 3M in personal communications (Chang 2020a;  Chang 2020b). Both oral and intravenous (IV) datasets were made available.

### TK modeling approach: a case study with PFOS and PFOA

We used the following stepwise approach to estimate human model parameters in our adapted Wambaugh model for PFOS and PFOA:

- We first estimated an identifiable set of sensitive parameters of the PFOA and PFOS Wambaugh model to fit the experimental data on nonhuman primates.
- We scaled the reparametrized Wambaugh model from Part I to humans using human values for the bodyweight (70 kg) and cardiac output (12.5 L/h/kg^0.74^) following the procedure of Loccisano et al. (2011);
- We then adjusted the maximum rate of the saturable resorption parameter (*T_m_*, mg/h) to match the half-life estimated in humans from an exposure study deemed to be representative of the general population (Li et al. 2018).

### Inferences

The Wambaugh model was coded and studied in R using the mrgsolve package framework and the FME package (Soetaert and Petzoldt 2010; Baron 2023) for model adjustment. Further details are provided in the R code (Section S1).

Not all the parameters were able to be independently estimated from the available data, as evident in the large credible intervals reported in Wambaugh et al. (2013). The number of identifiable parameters was first determined herein using the “collin” function from the FME package (Soetaert and Petzoldt 2010). An iterative process was then applied, through the analysis of the convergence and the outputs of Monte-Carlo Markov Chain (MCMC) inference processes (modMCMC function from FME), to further check the identifiability of the model. When a parameter could not be independently estimated, a value was assumed based on that reported in the literature or using an optimized value for other compounds as shown in the parameter tables. The initial parameter values for estimation are shown in Table S1. An iterative process was applied from these starting values as needed to minimize the sum of squared residuals. Most of these starting values were consistent with the optimized parameter values from Wambaugh et al. (2013); however, the values for *Free*, the proportion of free compound in the central compartment, were updated to reflect the most current assessment of the fraction of unbound compound in human plasma (Smeltz et al. 2023). Additionally, a parameter for bioavailability was added to the model to align with the Verner et al. (2016) model implemented by the US EPA (2024a, 2024b) in their final reference dose level setting for PFOS and PFOA. The bioavailability was initially set at 90% for both compounds to reflect a high fraction of an administered dose reaching systemic circulation after oral ingestion; however, for PFOA, the parameter was found to be identifiable and estimable from the data for the nonhuman primate model only. The 95% credible intervals for the estimated parameter values were determined from the output of the MCMC.

### Model checking and validation

Model performance was evaluated with mean square log errors (MSLE) and by visually comparing the commonalities between the observed and predicted data. Predictions that were on average within a factor of 2 of the experimental data were considered adequate (World Health Organization 2010). For the human models specifically, the performance of the resulting models was assessed by (i) simulating biomonitoring data in the NHANES and (ii) predicting point of departure human equivalent doses (POD_HED_).

For validation with NHANES biomonitoring data, the observed PFOS and PFOA serum levels were extracted from the NHANES data files (1999 to 2000, 2003 to 2004 and 2017 to 2018 surveys) with the respective date of birth. Data for individuals <20 years of age were excluded from the fitting process because the TK model does not include a development compartment and thus underestimates serum levels in children and young adults by excluding gestation and breastfeeding exposures. Similarly, individuals ≥80 years of age were excluded as their exact age is not reported in NHANES. For each survey year and each age (21 to 79 years), the mean serum concentration of each PFAS was estimated using Centers for Disease Control and Prevention’s recommended procedure (Mirel et al. 2013; Chen et al. 2018, 2020) with the R survey package (Lumley 2004; Lumley et al. 2023). An exposure model was estimated based on the methodology and results obtained in Wong et al. (2014) for each compound, i.e. with PFAS exposure varying log-linearly with calendar time, starting in 1950 and with discontinuous breaks in 1990 and 1998. This time trend represents the increasing use of these chemicals from 1950 to 1990, followed by a decrease in their use and a transition to more restricted use after 1998 (Wong et al. 2014; Dzierlenga et al. 2020). The level of exposure in 1990, 1998, and 2017 were estimated using the FME modFit function. PFOS and PFOA serum levels in each survey were simulated using the corresponding modeled exposure to PFAS and the human-adapted models presented herein. The predicted and observed serum levels were compared to ensure the proposed approach yielded models capable of capturing available biomonitoring trends.

To further evaluate the utility of the approach for decision-making purposes, the resulting TK models were used to calculate the POD_HED_ for points of departure for cardiovascular and hepatic endpoints selected by the EPA in the Final Human Health Toxicity Assessments for PFOA and PFOS (US EPA 2024a, 2024b). As the model developed herein does not account for pregnancy or breastfeeding, developmental and immunological effects were not selected for evaluation.

### Application to other compounds

Nonhuman primate data were also shared for PFBS and PFHxS (Chang 2020c; Chang 2020d) and available in Sundström et al. (2012) and Olsen et al. (2009). Given the short half-life of PFBS (<7 days) and the likely minimal relevance of the saturable resorption process, we decided not to pursue the study of this compound further. The adapted Wambaugh model was thus only further parameterized for PFHxS following the procedure outlined in sections “Toxicokinetic modeling approach: a case study with PFOS and PFOA,” “Inferences,” and “Model checking and validation”. The starting values for the parameter estimation for PFHxS were the same as the starting values used for PFOS in Table S1.

## Results

### Model parameters estimated for humans for both PFOA and PFOS

The Wambaugh model was fitted to the available nonhuman primate data for PFOS (Seacat et al. 2002; Chang et al. 2012) and PFOA (Butenhoff et al. 2004). Of the 8 chemical-specific parameters in the model (*bioAv*, *VCC*, *Tmc*, *K_t_, Free*, *k_12_*, *k_21_*, and *k_a_*), 6 and 8 parameters were a priori identifiable for PFOA and PFOS, respectively, from the “collin” function. However, further MCMC tries clearly indicated additional collinearities in the estimates. As such, the proportion of free compound in the serum (*Free*), the rate of absorption from the gut (*k_a_*), and the rate to and from the second compartment (*k_12_*, *k_21_*) were assigned set values from the iterative evaluation of the starting parameters (Table S1). The impact of parameter changes was evaluated with a sensitivity analysis.

The *VCC*, *Tmc*, *K_t_*, and *bioAv* (PFOA) parameters were estimated from the data. The mean and MCMC 95% credible interval for each parameter are shown in Table 1. Changing the value of *k_a_*, *k_12_*, or *k_12_* by up to 2 orders of magnitude resulted in an average change in value for *VCC*, *Tmc*, *K_t_*, and *bioAv* (PFOA) of less than 10% (Tables S2 and S3), suggesting a robustness of the inference process given the available data. Increasing the ratio *k_12_*/*k_21_* to values equal to or greater than 100 resulted in changes in *VCC* of greater than 10%, as noted in Wambaugh et al. (2013). The *Free* parameter was found to be highly correlated to the resorption parameters; changing *Free* will then result in a change in *Tmc* and *K_t_*.

**Table 1.** Mean (95% CI) TK parameters for PFOS and PFOA fit to nonhuman primate data and scaled to humans.

Species	Type of variable	Variable	Name	Mean (95% CI)		Source
				PFOS	PFOA	
Nonhuman primate						
	Physiological	Body weight (kg)	*BW*	5^a^	4.1^a^	Butenhoff et al. (2004); Chang et al. (2012); Seacat et al. (2002)
		Cardiac blood output (L/h/kg^0.74^)	*QCC*	19.8	19.8	Wambaugh et al. (2013)
		Fraction of cardiac output to filtrate	*QfilC*	0.15	0.15	Wambaugh et al. (2013)
		Volume of filtrate compartment (L/kg)	*VfilC*	4.00E-04	4.00E-04	Loccisano et al. (2011)
	Chemical-specific	Bioavailability	*bioAv*	0.9	0.32 (0.20, 0.46)	US EPA (2024a) (PFOS) Optimized (PFOA)
		Volume of distribution central compartment (L/kg)	*VCC*	0.24 (0.24, 0.25)	0.23 (0.18, 0.29)	Optimized
		Saturable resorption rate (mg/h/kg)	*Tmc*	2.5 (2.31, 2.80)	0.28 (0.17, 0.53)	Optimized
		Saturable resorption affinity (mg/L)	*K_t_*	0.004 (0.003, 0.004)	0.017 (0.010, 0.041)	Optimized
		Proportion of free compound in serum	*Free*	4.50E-03	1.20E-3	Smeltz et al. (2023)
		Rate from central to second compartment (1/h)	*k_12_*	3.3	3.3	Andersen et al. (2006)
		Rate from second to central compartment (1/h)	*k_21_*	3.4	3.4	Andersen et al. (2006)
		Rate from gut to central compartment (1/h)	*k_a_*	132	230	Wambaugh et al. (2013)
Human						
	Physiological	Body weight (kg)	*BW*	70	70	Loccisano et al. (2011)
		Cardiac blood output (L/h/kg^0.74^)	*QCC*	12.5	12.5	Loccisano et al. (2011)
	Chemical-specific	Bioavailability	*bioAv*	0.9	0.9	US EPA (2024a, 2024b)
		Saturable resorption rate (mg/h/kg)	*Tmc*	1.07	0.54	Optimized
		Saturable resorption affinity (mg/L)	*K_t_*	0.004	0.008^b^	Optimized (nonhuman primate)
		Half-life (years)	*T_1/2_*	3.4	2.7	Li et al. (2018)

a The code uses the average body weight for each nonhuman primate or group of nonhuman primates as reported in the original studies.

b If the bioavailability is set to 90% for the nonhuman primate model of PFOA, the *Tmc* and *K_t_* values for the nonhuman primates are 0.15 mg/h/kg and 0.008 mg/L, respectively. For the human model, a set value of 0.008 mg/L for *K_t_* was used as it is representative of the higher bioavailability considered more typical for intake of food and water.

The animal models were scaled to humans by adjusting the cardiac blood output (*QCC*) to 12.5 L/h/kg^0.74^ and fitting a saturable resorption rate (*Tmc*) for a half-life of 3.4 years and 2.7 years for PFOS and PFOA, respectively (Li et al. 2018; Pizzurro et al. 2019). The remaining parameters were held constant between the animal and human models, as shown in Table 1, with the exception of the PFOA *bioAv* and *K_t_* parameters, which were refit to the nonhuman primate data assuming a bioavailability of 90% for application in the human model, as the lower bioavailability observed in the nonhuman primate model was assumed to be the result of experiment-specific conditions. The resulting values for *Tmc* were 1.07 and 0.54 mg/h/kg for PFOS and PFOA, respectively.

### Model validation

#### Animal models

**Fig. 2.**
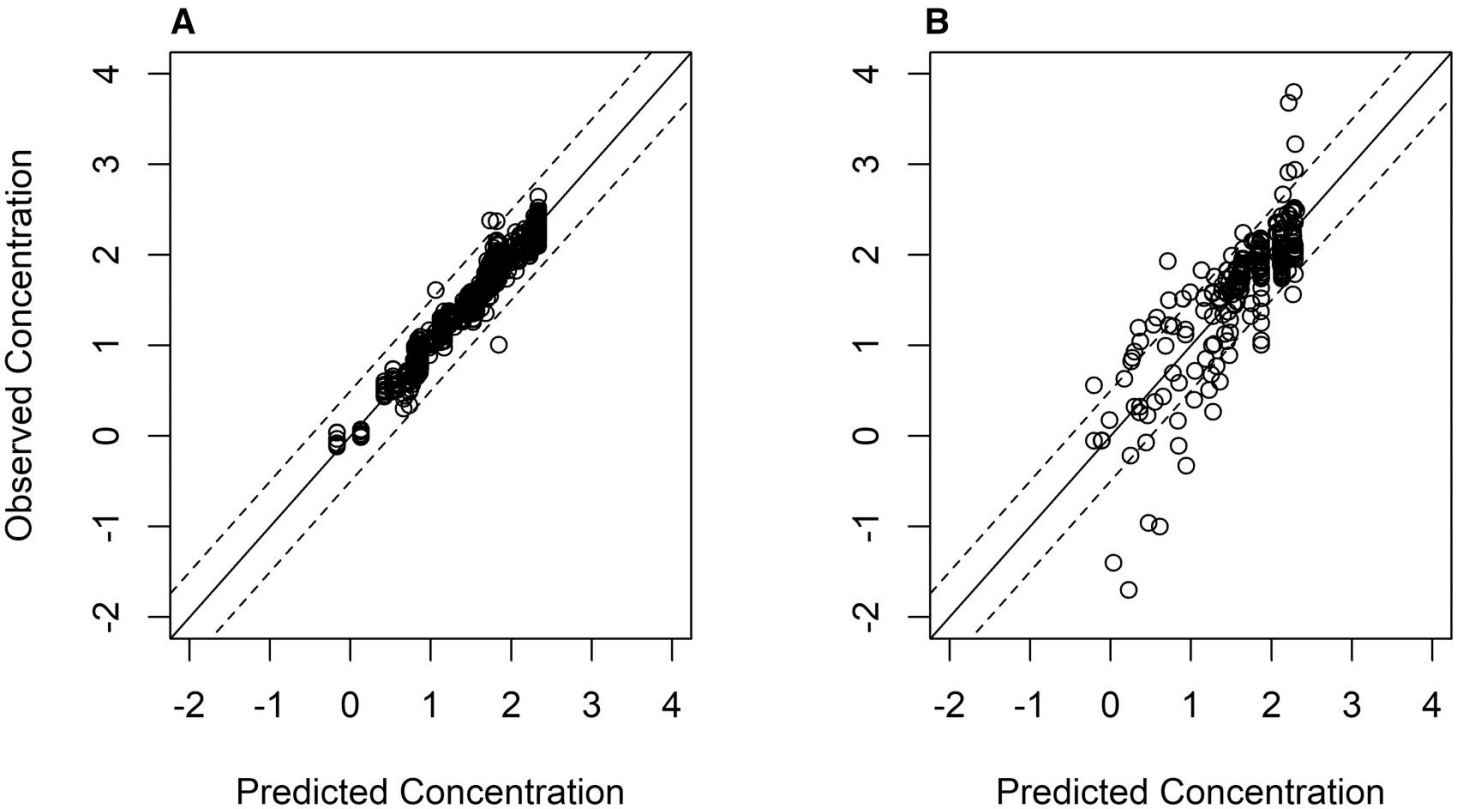
Model predictions for training data of serum concentrations (log_10_ µg/mL) of a) PFOS and b) PFOA in cynomolgus monkeys; dashed lines represent ± one-half log_10_.

Model performance was first evaluated by visually comparing model predictions to observed data (Fig. 2). The MSLE for the PFOS and PFOA models were 0.07 and 0.58, respectively, which fall within the range of MSLEs deemed acceptable by the EPA for the mice and rat data used to train and test the Wambaugh model (US EPA 2024a, 2024b). For PFOS, the model predictions were within a factor of 2 of the observed serum levels for 98% of the data points, whereas for PFOA, the model predictions were within a factor of 2 of the observed serum levels for 71% of the data. Models more closely approximated the serum levels during and after repeated oral exposures to PFOS and PFOA, than the serum levels after singular IV exposures (Figs S1 to S4) (US EPA 2024a, 2024b).

#### Human models—simulating biomonitoring data

**Fig. 3.**
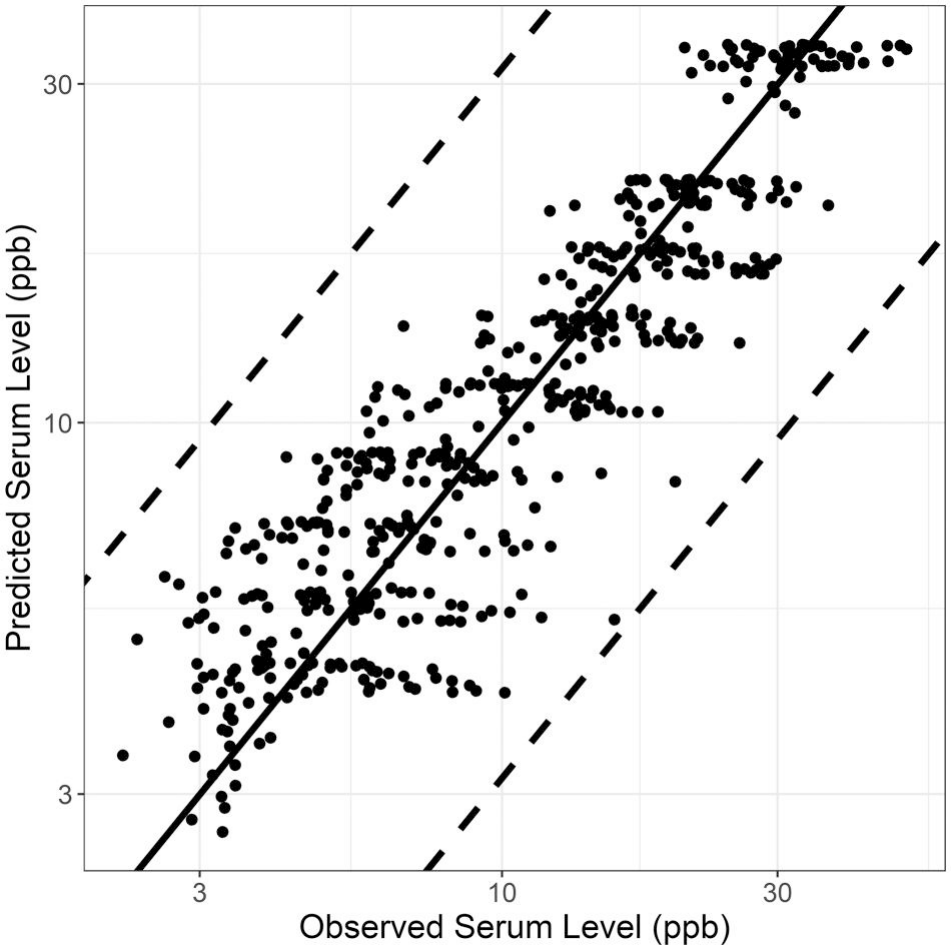
Observed and predicted PFOS serum levels (ppb) for NHANES 1999 to 2017; dashed lines represent ± one-half log_10_.

**Fig. 4.**
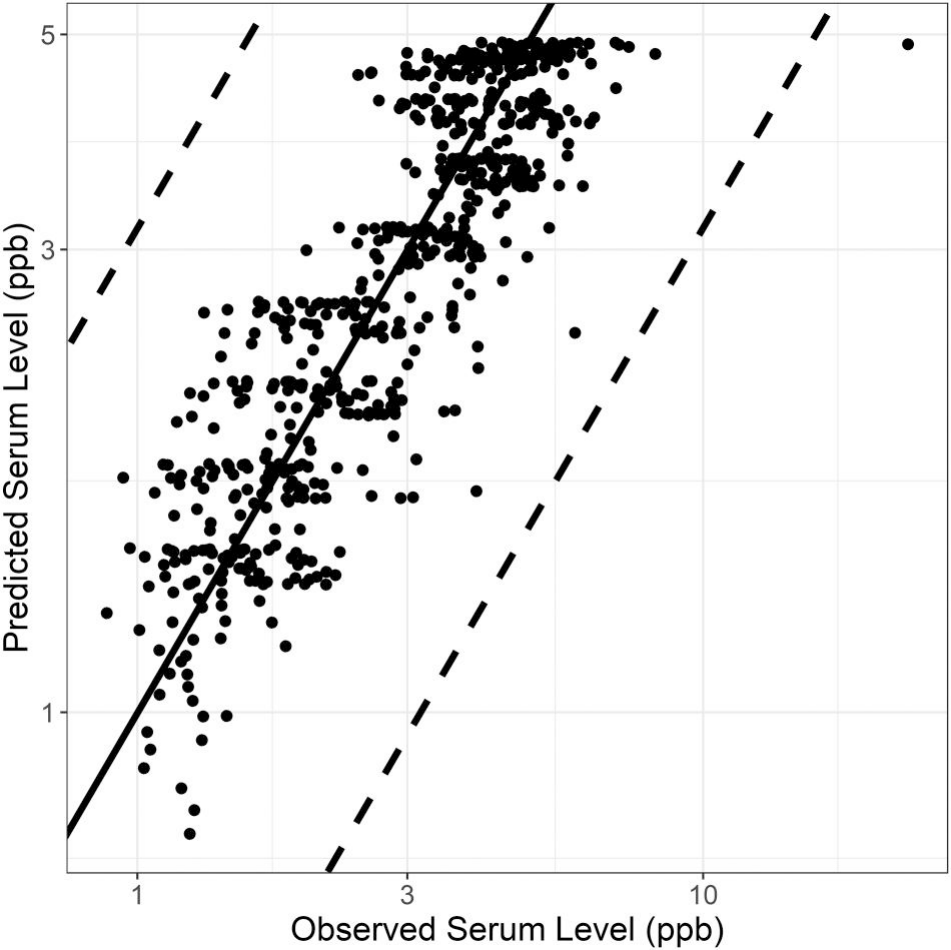
Observed and predicted PFOA serum levels (ppb) for NHANES 1999 to 2017; dashed lines represent ± one-half log_10_.

Mean parameter estimates for the human PFOS and PFOA models were used to simulate NHANES biomonitoring data. The PFOS model predicted serum levels with an MSLE of 0.07 (Fig. 3) and an underlying exposure model that peaked in 1990 at 14.9 ng PFOS/kg BW/day, fell to 2.5 ng PFOS/kg BW/day in 1998, and continued decreasing to 0.24 ng PFOS/kg BW/day in 2017 (Fig. S5). The PFOA model more closely predicted the NHANES observed serum levels with an MSLE of 0.03 (Fig. 4) and an underlying exposure model that increased from 0.45 ng/kg BW/day in 1990 to 1.1 ng/kg BW/day in 1998 and fell to 0.13 ng/kg BW/day in 2017 (Fig. S6). The models were able to match the trends for change in PFOS (Fig. S7) and PFOA (Fig. S8) serum levels for individuals born in the 1940s through 1990s. Notably, including variability and uncertainty in the parameter estimates for simulating the biomonitoring data would capture even more of the observed data (Figs S7 and S8).

#### Comparisons to the EPA-modified Verner model

The human models were also used to calculate the POD_HED_ for the cardiovascular and hepatic endpoints evaluated in the EPA’s Human Health Toxicity Assessments for PFOS and PFOA. The POD_HEDs_ calculated at steady state for a 70 kg individual using the model presented in this study were approximately 1.5 times greater than those calculated with the Verner model (US EPA 2024a, 2024b) as shown in Table 2. The differences at doses below saturation arise from the faster clearance predicted by the human models developed herein compared with the clearance in the EPA-modified Verner model.

**Table 2.** Comparing the POD_HEDs_ calculated with the Verner model in (US EPA 2024a, 2024b) for PFOS and PFOA to estimates calculated with the human-adapted Wambaugh model.

Compound	Effect	Outcome	Study	POD (mg/L)	EPA’s POD_HED_ (mg/kg/day)	Our POD_HED_ (mg/kg/day)
PFOS	Cardiovascular	Increased total cholesterol	Dong et al. (2019)	0.009	1.20E-06	1.43E-06
			Steenland et al. (2009)	0.010	1.22E-06	1.45E-06
			Lin et al. (2019)	0.067	8.51E-06	1.02E-05
	Hepatic	Elevated ALT	Gallo et al. (2012)	0.057	7.27E-06	8.68E-06
			Nian et al. (2019)	0.015	1.94E-06	2.31E-06
		Increased individual cell necrosis	Butenhoff et al. (2012)	27	3.45E-03	4.43E-03
PFOA	Cardiovascular	Increased total cholesterol	Dong et al. (2019)	0.002	2.75E-07	4.15E-07
			Steenland et al. (2009)	0.004	5.10E-07	7.70E-07
			Lin et al. (2019)	0.005	6.34E-07	9.57E-07
	Hepatic	Elevated ALT	Gallo et al. (2012)	0.02	2.15E-06	3.25E-06
			Darrow et al. (2016)	0.07	7.92E-06	1.20E-05
			Nian et al. (2019)	0.004	4.51E-07	6.82E-07
		Increased focal necrosis	Loveless et al. (2008)	10	1.20E-03	1.84E-03
		Increased individual cell necrosis	Loveless et al. (2008)	36	4.32E-03	6.86E-03
		Increased hepatocyte single cell death	NTP (2020)	100	1.20E-02	2.10E-02
		Increased necrosis	NTP (2020)	26.9	3.23E-03	5.06E-03

### Application to PFHxS

After validating the described approach, a human model for PFHxS was developed using available nonhuman primate data (Chang 2020d). Only IV data were available for PFHxS, and thus the same chemical-specific parameters (*bioAv, Free*, *k_12_*, and *k_21_*) were held constant in the model as for the previous compounds in addition to the rate of absorption from the stomach (*k_a_*). For PFHxS, *Tmc* and *K_t_* were also determined to be too highly correlated to be estimated independently. The *k_a_* and *K_t_* parameters were assumed to be similar to those for PFOS (Table 3), and *VCC* and *Tmc* were fitted to the data. The optimized model parameters are shown in Table 3, and the final model predictions for the nonhuman primate model were within a factor of 2 of the observed serum levels for 86% of the data points, with an MSLE of 0.17 (Figs S9 and S10). The animal model was scaled to human using a half-life of 5.3 years, resulting in a *Tmc* of 0.47 mg/h/kg (Li et al. 2018). Notably, for doses well below saturation (<1000 mg/kg BW/day), the human model is associated with a clearance of approximately 0.06 mL/kg-day for a 70 kg adult, which falls between the clearance values applied for males and females outside of reproductive ages (0.041 mL/kg-day) and females of reproductive ages (0.072 mL/kg-day) in the EPA’s derivation of PFHxS candidate toxicity values (US EPA 2025).

**Table 3.** Optimized TK parameters and 95% CI for nonhuman primate and human models of PFHxS.

Species	Type of variable	Variable	Name	Mean (95% CI)	Source
Nonhuman primate					
	Physiological	Body weight (kg)	*BW*	5	Chang (2020d)
		Cardiac blood output (L/h/kg^0.74^)	*QCC*	19.8	Wambaugh et al. (2013)
		Fraction of cardiac output to filtrate	*QfilC*	0.15	Wambaugh et al. (2013)
		Volume of filtrate compartment (L/Kg)	*VfilC*	4.00E-04	Loccisano et al. (2011)
	Chemical-specific	Bioavailability	*bioAv*	0.9	Assumed (PFOS)
		Volume of distribution central compartment (L/kg)	*VCC*	0.17 (0.16, 0.20)	Optimized
		Saturable resorption rate (mg/h/kg)	*Tmc*	0.21 (0.18, 0.25)	Optimized
		Saturable resorption affinity (mg/L)	*K_t_*	0.004	Assumed (PFOS)
		Proportion of free compound in serum	*Free*	0.0009	Smeltz et al. (2023)
		Rate from central to second compartment (1/h)	*k_12_*	3.3	Andersen et al. (2006)
		Rate from second to central compartment (1/h)	*k_21_*	3.4	Andersen et al. (2006)
		Rate from gut to central compartment (1/h)	*k_a_*	132	Wambaugh et al. (2013)
Human					
	Physiological	Body weight (kg)	*BW*	70	Loccisano et al. (2011)
		Cardiac blood output (L/h/kg^0.74^)	*QCC*	12.5	Loccisano et al. (2011)
	Chemical-specific	Saturable resorption rate (mg/h/kg)	*Tmc*	0.47	Optimized
		Half-life (years)	*T_1/2_*	5.3	Li et al. (2018)

## Discussion

We developed an approach that improves our ability to connect PFAS exposures to endpoints of concern for less studied PFAS compounds through the adaptation of published TK models for nonhuman primates for use in humans over the age of 20. Our results suggest that only a few key parameters (*BW*, *QCC*, *T_1/2_*) are needed to adapt the Wambaugh nonhuman primate compartment model to be useful for human inferences, minimizing the reliance on human-specific data for new TK model development. A nonhuman primate PBTK model was similarly adapted in Loccisano et al. (2011), and although PBTK models are generally preferred over compartment approaches, a compartment model-based approach is an attractive alternative herein considering the need for simplicity when conducting dosimetry calculations for PFAS compounds with minimal human TK data. The Wambaugh model was the foundation of our approach because of its simplicity and its demonstrated use in regulatory works (US EPA 2024a, 2024b). Although PBTK models have been developed for PFOA, PFOS, and PFHxS, and these models can estimate additional tissue concentrations beyond the serum, serum contains the major proportion of the compounds in question (Forsthuber et al. 2020). Furthermore, our knowledge of dietary exposure to PFAS is primarily informed by serum data (Calafat et al. 2007, 2019). Thus, the simplicity of a multi-compartment model for this approach makes it fit for use.

A specific effort was made in this study to identify correlations among parameters that would impair the inference process. These correlations can lead to extremely large credible intervals in the estimates of the MCMC inference process, as can be observed in Wambaugh et al. (2013). The parameters deemed identifiable were associated with much narrower credible intervals. Sensitivity analyses were conducted for the parameters held constant in the fitting process, and although some parameters (*k_12_, k_21_, k_a_*) were found to have a minimal impact on model outputs for the tested values (Tables S2 and S3), the values for *Free* and bioavailability for each compound were found to have a strong influence on results. The *Free* parameters in these models were selected based on protein binding assay findings (Smeltz et al. 2023). An advantage of this approach is the availability of compound-specific *Free* values for multiple PFAS compounds of interest. However, the binding efficacy may be higher in vitro than in vivo. Different *Free* values will correspond to different resorption parameters, and the model can be adjusted accordingly with newer findings. The bioavailability parameter directly impacts the amount of the compounds that enter the serum, and the default values were chosen to reflect the high absorption observed in animals after ingestion exposures (US EPA 2024a, 2024b). The parameter could be adjusted in future applications to reflect dosing scenario and experiment-specific conditions as needed.

Our approach was validated using NHANES biomonitoring data. Both the PFOS and PFOA TK models were able to simulate the observed trends in US serum concentrations between the 1940s and 2000s (Figs S7 and S8). In addition to evaluating the TK models’ ability to simulate general biomonitoring data trends over time, the feasibility of the underlying exposure models was also considered. Wong et al. (2014) assumed a constant exposure between 1990 and 1998, and the optimized intake was found to be 3.9 ng PFOS/kg BW/day. The optimized exposure model herein estimated a peak weight-based intake of 14 ng PFOS/kg BW/day in 1990 that fell to 2.5 ng PFOS/kg BW/day in 1998 for the same population. The earliest available serum data for both approaches was recorded in 1999, thus limiting the inferences that can be made about the weight-based intakes in previous years. Accordingly, the lower 1998 estimated intake herein may be due to the use of an exposure model that predicts higher exposures pre-1998, or the use of a TK model with a slower clearance rate compared with Wong et al. (2014). The estimated PFOA peak intake in 1998 of 1.1 ng PFOA/kg BW/day was also lower than previous estimates of approximately 6 ng PFOA/kg BW/day (Dzierlenga et al. 2020). This could also be explained by a slower clearance rate in our TK model compared with the Dzierlenga et al. (2020) model that included menstrual clearance and age-dependent glomerular filtration rates. Importantly, these comparisons highlight the variability in reverse dosimetry estimates using NHANES data that can arise from exposure and TK model selection.

We also calculated the POD_HED_ for the cardiovascular and hepatic endpoints evaluated in the EPA’s Human Health Toxicity Assessments to validate our approach. In that regard, our estimated human equivalent doses for the selected PODs were approximately 1.5 times greater than those estimated by the Verner model at steady state for a 70 kg individual (US EPA 2024a, 2024b). The differences arise from the higher volume of distribution estimated in our Wambaugh-adapted models compared with the one-compartment EPA-modified Verner model. If we consider the full spectrum of possible exposure doses, the EPA-modified Verner model would not be applicable for doses past the point of saturation, whereas the Wambaugh-adapted model incorporates saturable absorption and could be used. These comparisons suggest that the TK models developed with our approach are sufficiently similar to those applied for regulatory purposes, while being able to incorporate the more advanced saturable resorption mechanism, which increases the model’s utility for making inferences from animal datasets and extreme human exposure scenarios. The utility of the approach is further demonstrated by its application to PFHxS, which yielded a model with a clearance rate between those selected for males and females outside of reproductive ages and females of reproductive age in the EPA’s derivation of PFHxS candidate toxicity values (US EPA 2025).

This work demonstrates the feasibility of our approach to adapt TK models from nonhuman primates to humans with a level of accuracy. The application and continued development of this approach for PFAS compounds of interest will advance our ability to relate dietary PFAS to endpoints of concern. The inclusion of a development compartment to model gestational and lactational exposure to infants is a noted area for future improvement, as this is needed for baby and infant applications. Modifications were made to the Wambaugh animal model to account for gestation, lactation, and postweaning phases in other works (US EPA 2024a, 2024b), and the applicability of these modifications for our proposed approach to adapting nonhuman primate models for humans needs to be further explored. To our knowledge, there are limited additional nonhuman primate studies available in the literature for the wide range of PFAS compounds found in the diet (Olsen et al. 2009; Chang 2020c). Our framework could also be used to leverage data from other animal studies, with the determination of the necessary species-specific adaptations to the approach. Accommodating data from test animals with less physiological similarities to humans will likely require the scaling of additional parameters beyond those identified in the nonhuman primate–human framework. The results presented herein can be used to guide the determination of the species-specific parameters for scaling and to calibrate the final model forms.

## Supplementary Material

kfaf087_Supplementary_Data
