# Supplementary material for: Adapting existing toxicokinetic models to relate perfluoroalkyl and polyfluoroalkyl intake to biomarkers in humans
Source: Toxicol Sci. 2025 Jun 16;207(1):139–47. doi: 10.1093/toxsci/kfaf087 (PMC12448206; doi:10.1093/toxsci/kfaf087)
Supplement: kfaf087_Supplementary_Data [file kfaf087_supplementary_data.docx]

Supplementary Section

**Title:** Adapting Existing Toxicokinetic Models to Relate Perfluoroalkyl and Polyfluoroalkyl Intake to Biomarkers in Humans

**Authors**: Kara Dean^1^*, Régis Pouillot^2^, Jane Van Doren^1^, Sofia Santillana-Farakos^1^

^1^Human Foods Program, U.S. Food and Drug Administration, 5001 Campus Drive, College Park, MD 20740 USA.

^2^Consultant. 18 rue Mohamed Al Ghazi, Rabat 10170, Morocco

*kara.dean@fda.hhs.gov

**Contents:**

Table S1…………………………………………………………………………………………………….2

Table S2………………………………………………………………………………………………….....2

Table S3………………………………………………………………………………………………….....3

Figure S1…………………………………………………………………………………………………....4

Figure S2………………………………………………………………………………………………........5

Figure S3…………………………………………………………………………………………………....6

Figure S4………………………………………………………………………………………………........7

Figure S5………………………………………………………………………………………………........8

Figure S6………………………………………………………………………………………………........9

Figure S7………………………………………………………………………………………………......10

Figure S8………………………………………………………………………………………………......11

Figure S9………………………………………………………………………………………………......12

Figure S10………………………………………………………………………………………………....13

Section S1………………………………………………………………………………………………....14

**Table S1**: Initial TK Parameter Values for PFOS and PFOA Fit to Nonhuman Primate Data

| Variable | Name | Starting Value | | Source |
| --- | --- | --- | --- | --- |
|  |  | PFOS | PFOA |  |
| Body Weight (kg) | *BW* | 5* | 4.1* | Seacat et al., 2002; Chang et al., 2012; Butenhoff et al., 2004 |
| Bioavailability | *bioAv* | 0.9 | 0.9 | Assumed |
| Volume of distribution Central Compartment (L/kg) | *VCC* | 0.303 | 0.4 | Wambaugh et al. (2013) |
| Fraction of the filtrate compartment volume (L/Kg) | *VfilC* | 0.0004 | 0.0004 | Loccisano et al. (2011) |
| Saturable resorption rate (mg/h/kg) | *Tmc* | 15.5 | 3.9 | Wambaugh et al. (2013) |
| Saturable resorption affinity (mg/L) | *K_t_* | 0.00594 | 0.043 | Wambaugh et al. (2013) |
| Proportion of Free compound in the central compartment | *Free* | 0.0045 | 0.001 | Smeltz et al. (2023) |
| Rate of Flow from the central compartment to the second (1/h) | *k_12_* | 3.3 | 3.3 | Andersen et al. (2006) |
| Rate of Flow from the second compartment to the central (1/h) | *k_21_* | 3.4 | 3.4 | Andersen et al. (2006) |
| Rate from gut to central compartment (1/h) | *k_a_* | 132 | 230 | Wambaugh et al. (2013) |

*Average body weight for all specimens; the code uses the average body weight for each nonhuman primate or group of nonhuman primates as reported in the original studies

**Table S2**: Sensitivity Analysis of Changes in the PFOS Optimized Parameter Values (95% CI) for Various Combinations of *k_12_,* *k_2_*_1_, and *k_a_*

| Sensitivity Analysis Set | *bioAv* | *VCC*  (L/kg) | *Tmc* (mg/h/kg) | *K_t_*  (mg/L) | *k_12_*  (1/h) | *k_21_*  (1/h) | *k_a_*  (1/h) | *Free* | SSR |
| --- | --- | --- | --- | --- | --- | --- | --- | --- | --- |
| Base | 0.90 | 0.24  (0.24, 0.25) | 2.66  (2.31, 2.80) | 0.0039  (0.003, 0.004) | 3.3 | 3.4 | 132 | 0.0045 | 8.7 |
| Change k_a_ (lower) | 0.90 | 0.24  (0.24, 0.25) | 2.61  (2.31, 2.79) | 0.0038  (0.003, 0.004) | 3.3 | 3.4 | 10 | 0.0045 | 8.7 |
| Change k_a_ (higher) | 0.90 | 0.24  (0.24, 0.25) | 2.66  (2.29, 2.79) | 0.0039  (0.003, 0.004) | 3.3 | 3.4 | 500 | 0.0045 | 8.7 |
| Change k_a_ (higher, 2 orders) | 0.90 | 0.24  (0.24, 0.25) | 2.53  (2.30, 2.79) | 0.0036  (0.003, 0.004) | 3.3 | 3.4 | 13200 | 0.0045 | 8.7 |
| Change k_12_, k_21_ (ratio < 100) | 0.90 | 0.23  (0.23, 0.24) | 2.60  (2.30, 2.77) | 0.0038  (0.003, 0.004) | 330 | 34 | 132 | 0.0045 | 8.7 |
| Change k_12_, k_21_ (ratio < 100), and k_a_ (higher) | 0.90 | 0.23  (0.23, 0.24) | 2.61  (2.30, 2.76) | 0.0038  (0.003, 0.004) | 3.3 | 0.3 | 500 | 0.0045 | 8.7 |
| Change k_12_, k_21_ (ratio = 100) | 0.90 | 0.17  (0.16, 0.17) | 2.65  (2.31, 2.77) | 0.0039  (0.003, 0.004) | 100 | 1 | 132 | 0.0045 | 8.7 |
| Change k_12_, k_21_ (ratio = 100), and k_a_ (lower) | 0.90 | 0.17  (0.17, 0.18) | 2.63  (2.27, 2.74) | 0.0039  (0.003, 0.004) | 3.3 | 0.03 | 10 | 0.0045 | 8.4 |

*SSR=Sum of Squared Residuals

**Table S3**: Sensitivity Analysis of Changes in the PFOA Optimized Parameter Values (95% CI) for Various Combinations of *k_12_*, *k_21_*, and *k_a_*

| Sensitivity Analysis Set | *bioAv* | *VCC*  (L/kg) | *Tmc* (mg/h/kg) | *K_t_*  (mg/L) | *k_12_*  (1/h) | *k_21_*  (1/h) | *k_a_*  (1/h) | *Free* | SSR* |
| --- | --- | --- | --- | --- | --- | --- | --- | --- | --- |
| Base | 0.32  (0.20, 0.46) | 0.23  (0.19, 0.29) | 0.28  (0.17, 0.53) | 0.0172  (0.01, 0.04) | 3.3 | 3.4 | 132 | 0.0012 | 45.3 |
| Change k_a_ (lower) | 0.31  (0.18, 0.48) | 0.23  (0.19, 0.29) | 0.28  (0.17, 0.64) | 0.0176  (0.01, 0.05) | 3.3 | 3.4 | 10 | 0.0012 | 45.3 |
| Change k_a_ (higher) | 0.32  (0.19, 0.48) | 0.23  (0.19, 0.29) | 0.27  (0.18, 0.50) | 0.0172  (0.01, 0.04) | 3.3 | 3.4 | 500 | 0.0012 | 45.3 |
| Change k_a_ (higher, 2 orders) | 0.32  (0.19, 0.51) | 0.23  (0.19, 0.29) | 0.28  (0.17, 0.68) | 0.0172  (0.01, 0.05) | 3.3 | 3.4 | 13200 | 0.0012 | 45.3 |
| Change k_12_, k_21_ (ratio < 100) | 0.32  (0.20, 0.49) | 0.23  (0.19, 0.29) | 0.28  (0.17, 0.55) | 0.0172  (0.01, 0.04) | 330 | 34 | 132 | 0.0012 | 45.3 |
| Change k_12_, k_21_ (ratio < 100), and k_a_ (higher) | 0.32  (0.18, 0.47) | 0.23  (0.18, 0.29) | 0.27  (0.18, 0.64) | 0.0168  (0.01, 0.04) | 3.3 | 0.3 | 500 | 0.0012 | 45.3 |
| Change k_12_, k_21_ (ratio = 100) | 0.32  (0.19, 0.49) | 0.21  (0.16, 0.26) | 0.27  (0.16, 0.60) | 0.0169  (0.01, 0.04) | 100 | 1 | 132 | 0.0012 | 45.3 |
| Change k_12_, k_21_ (ratio = 100), and k_a_ (lower) | 0.32  (0.15, 0.48) | 0.21  (0.16, 0.27) | 0.26  (0.17, 1.12) | 0.0166  (0.01, 0.08) | 3.3 | 0.03 | 10 | 0.0012 | 44.8 |

*SSR=Sum of Squared Residuals


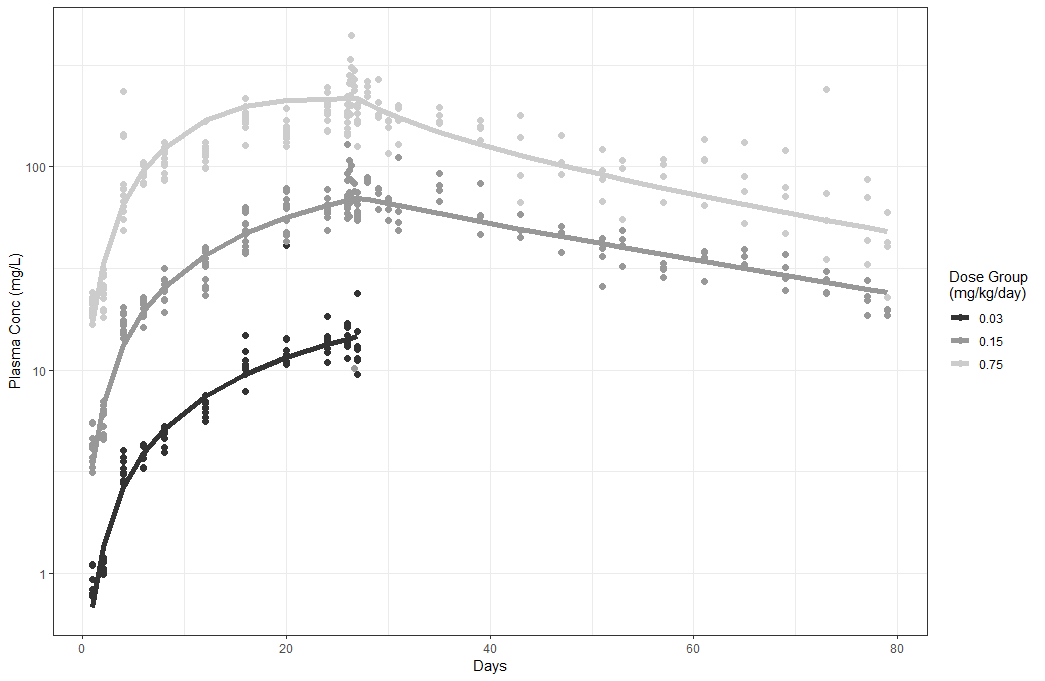
Figure S1: Observed (points) and predicted (lines) concentrations of PFOS in the plasma of nonhuman primates given daily oral doses of PFOS for 26 weeks


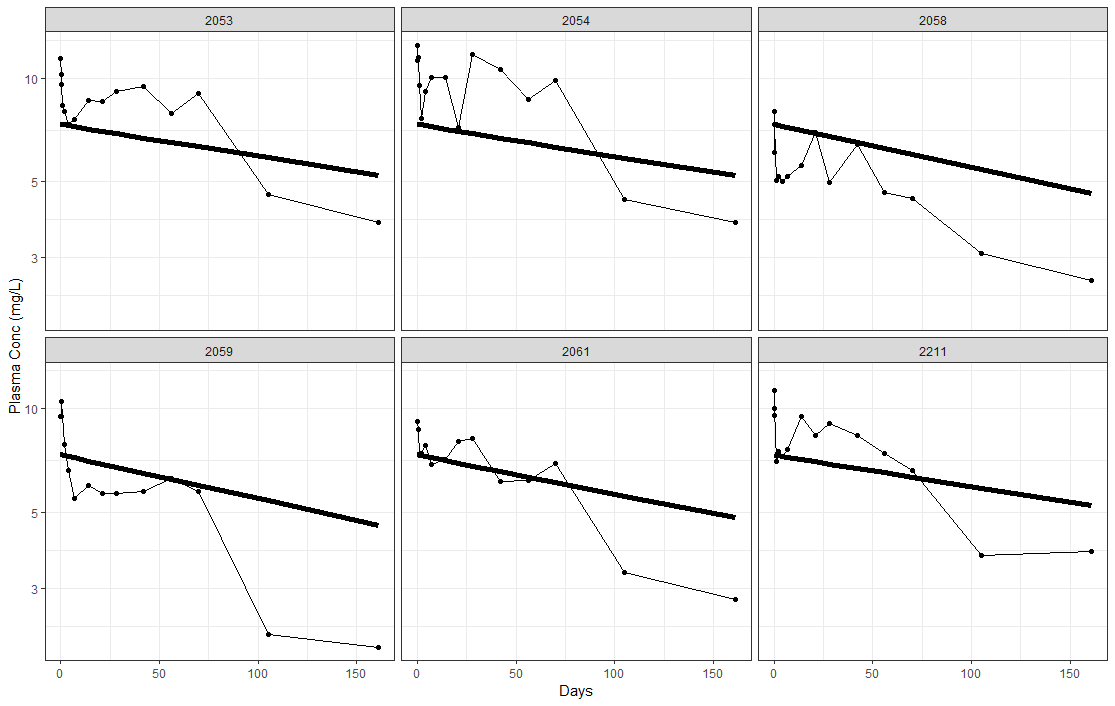
Figure S2: Observed (points with line) and predicted (bold line) concentration of PFOS in plasma of nonhuman primates after receiving a single intravenous dose of 2 mg/kg of PFOS


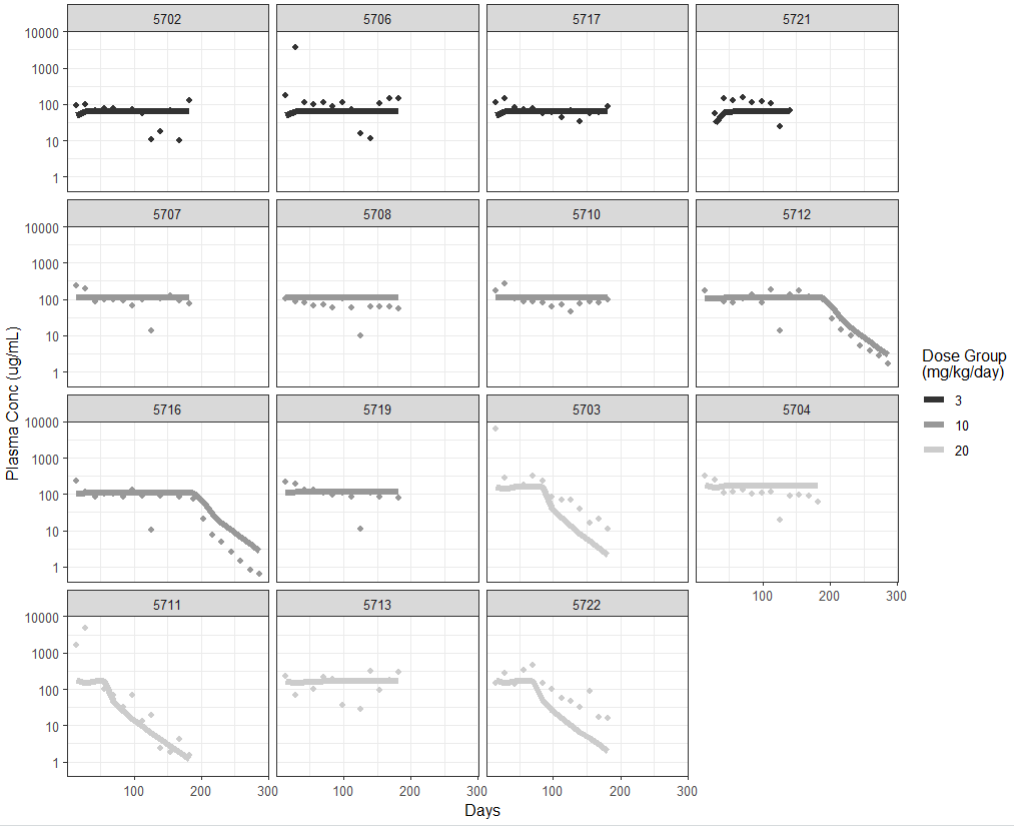
Figure S3: Observed (points) versus predicted (lines) concentrations of PFOA in the plasma of nonhuman primates while receiving daily oral doses of PFOA for six months


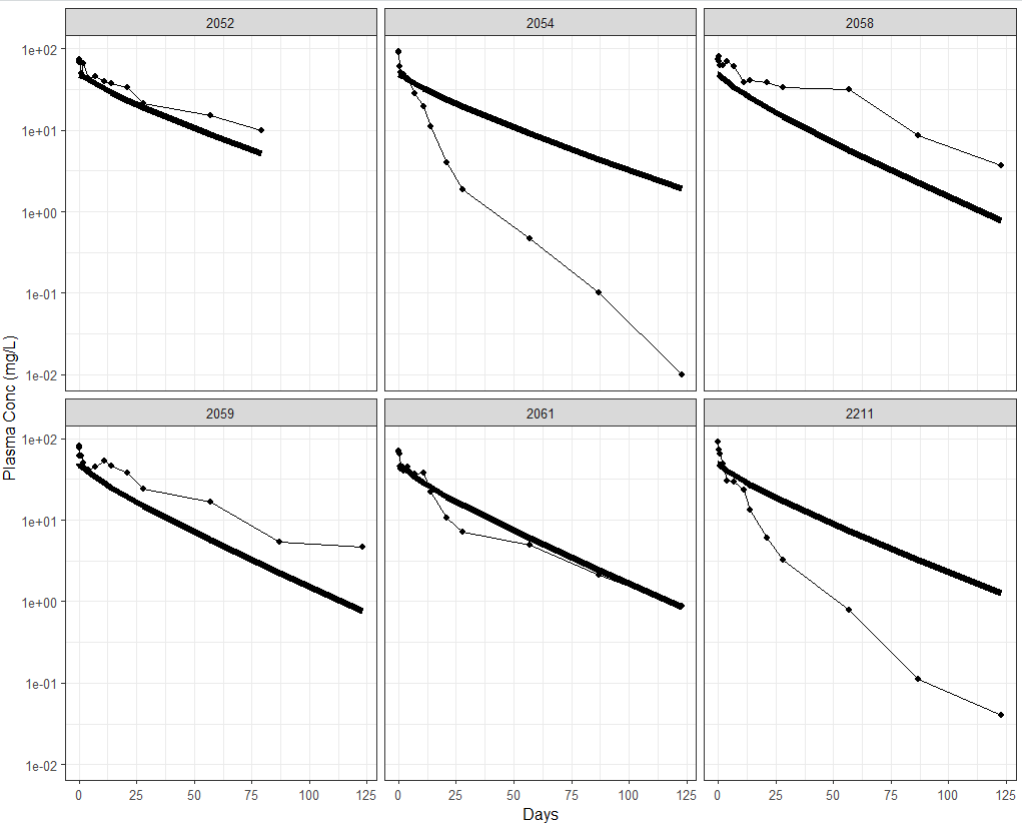
**Figure S4:** Observed (points) and predicted (lines) concentration of PFOA in plasma of nonhuman primates after receiving a single intravenous dose of 10 mg/kg of PFOA


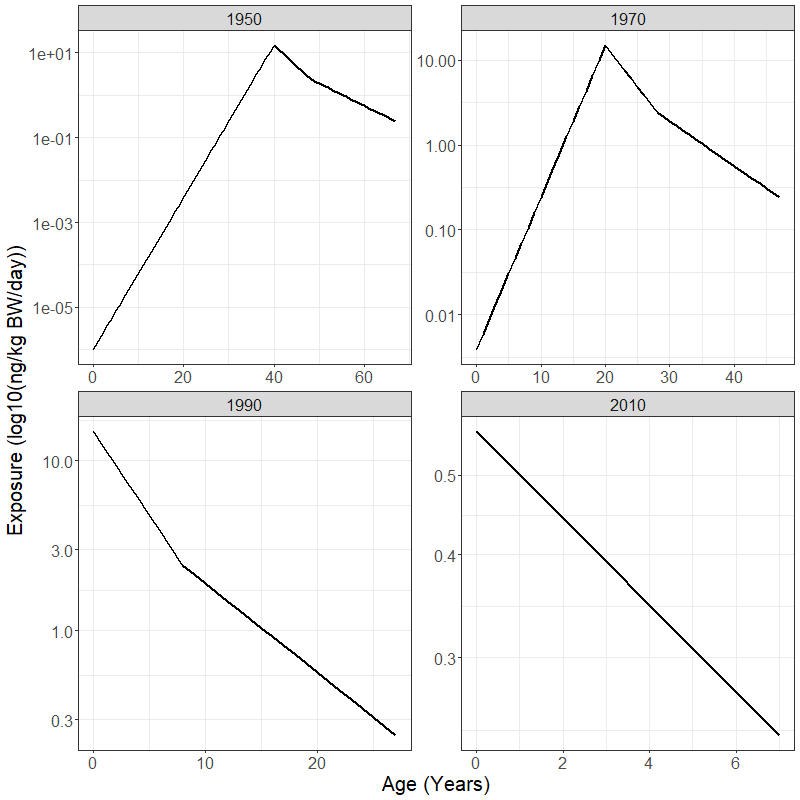


**Figure S5**: Exposure to PFOS for an average individual born in 1950, 1970, 1990 or 2010, as estimated by reverse dosimetry from NHANES data


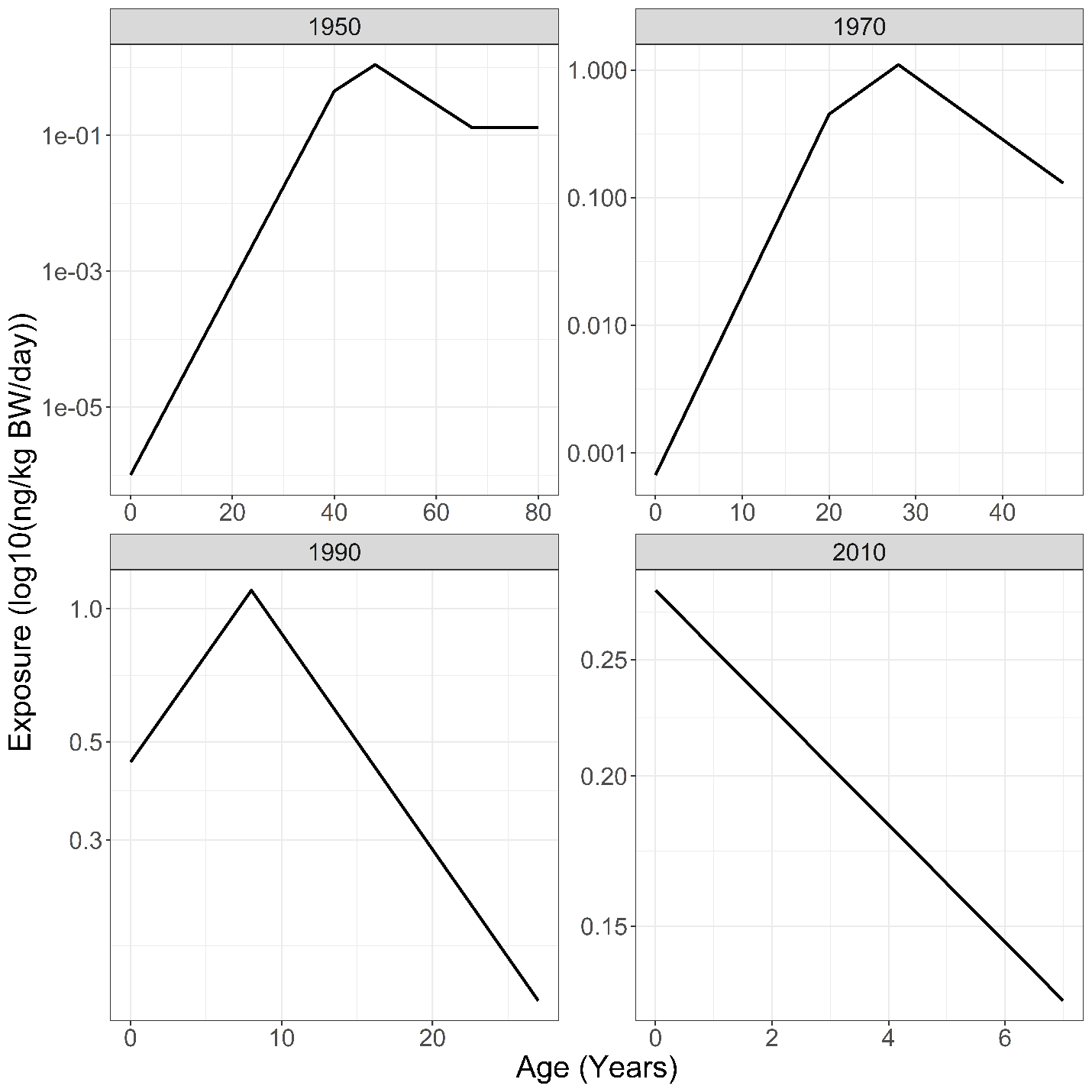


**Figure S6**: Exposure to PFOA for an average individual born in 1950, 1970, 1990 or 2010, as estimated by reverse dosimetry from NHANES data


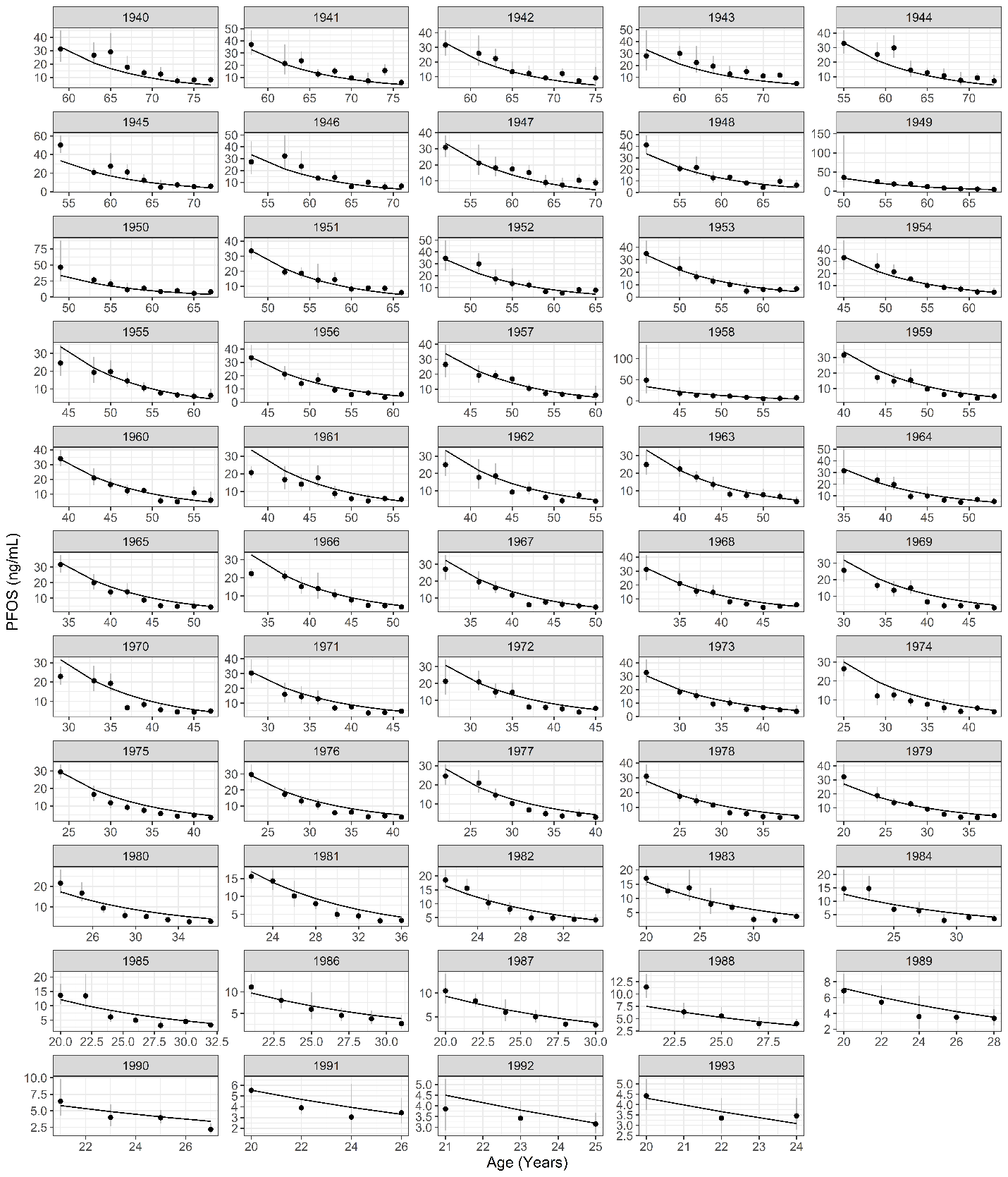


**Figure S7:**  Observed (points) and predicted (line) concentrations of PFOS through time for individuals born in 1940-1999


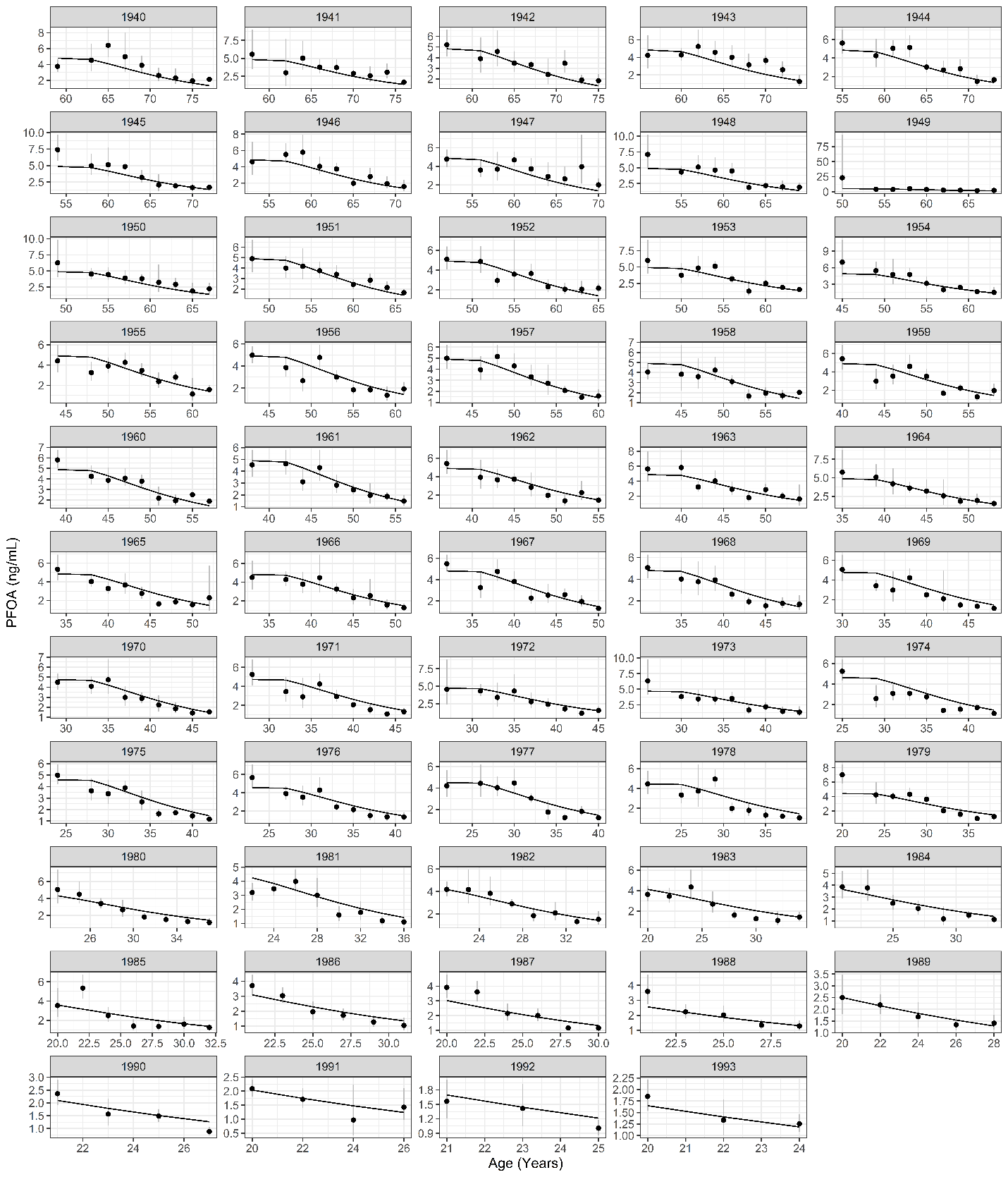


**Figure S8**: Observed (points) and predicted (line) concentrations of PFOA through time for individuals born in 1940-1999


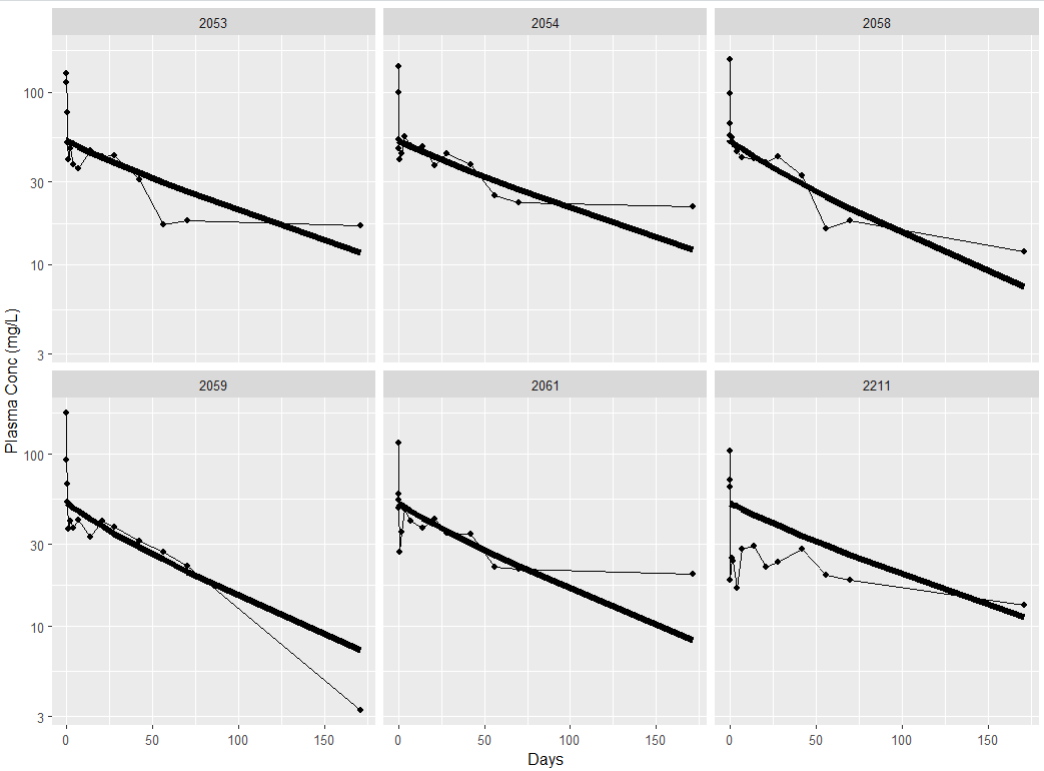
 **Fig S9**: Observed (points) and predicted (lines) concentration of PFHxS in plasma of nonhuman primates after receiving a single intravenous dose of 10 mg/kg of PFHxS

###
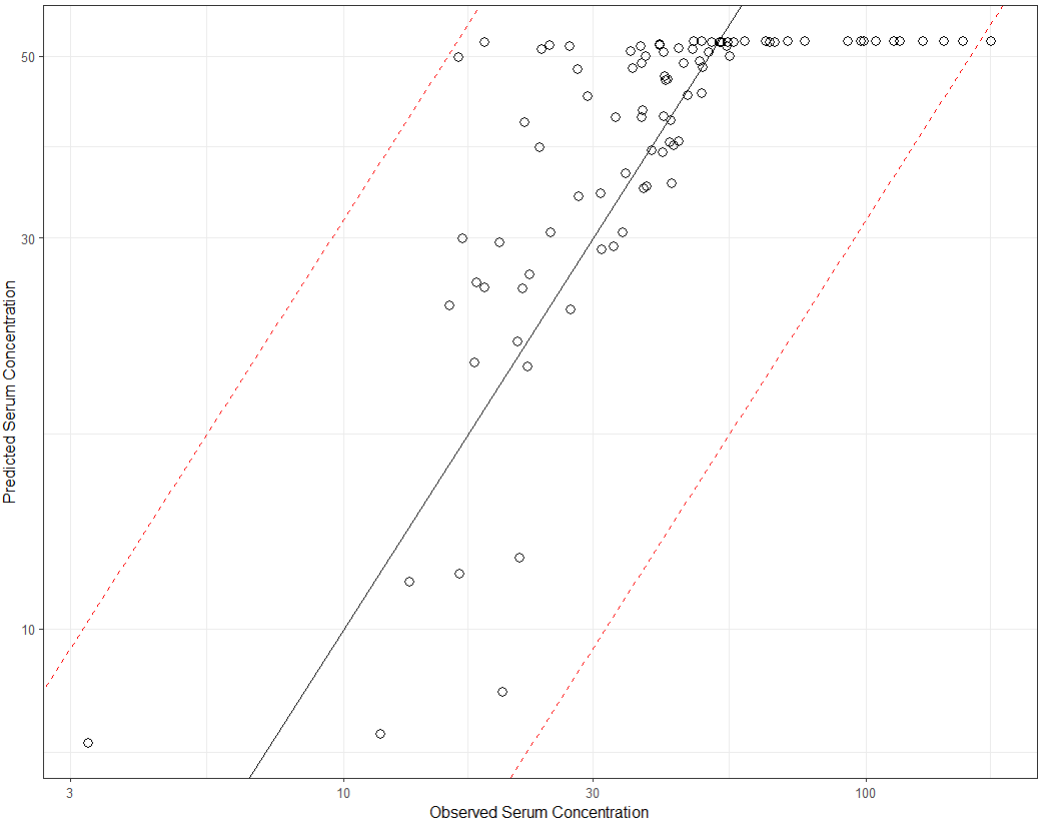
 **Figure S10**: Model predictions for training data of serum concentrations (µg/mL) of PFHxS in cynomolgus monkeys; dashed lines represent +/- one-half log10

**Section S1**: R mrgsolve model (PFOS)- Detailed code is available upon request to the corresponding author.

/*

* This model is based on the supplemental material from

* Wambaugh et al, 2013

* (mrgsolve model)

*/

$PROB

# Model: `Wambaugh 2013 monkey`

- Date: `r Sys.Date()`

- Version: 1.0

$PARAM @annotated // Parameter Declaration

BW : 4.5 : BW kg (will be specifically set through data)

bioAv : 0.9 : bioavailability in the Gut.

// Physiological parameters

// fractional blood flows

QCC : 19.8 : Cardiac blood output (L/h/kg^0.74)

QfilC : 0.15 : Fraction cardiac output going to filtrate (Kidney), Renal Blood Flow

// fractional tissue volumes

VfilC : 0.0004 : Fraction filtrate compartment volume (10% of kidney volume)(L/kg)

VCC : 0.303 : Volume of distribution (L/kg) (to be estimated)

// Chemical-specific parameters (PFOS)

Tmc : 15.5 : Maximum resorption rate (mg/h/kg) (to be estimated)

Kt : 0.00594 : Resorption affinity (mg/L) (to be estimated)

Free : 0.0045 : Free fraction of PFAS in plasma

k12 : 3.3 : From Anderson et al 2006 (/h)

k21 : 3.3 : From Anderson et al 2006 (/h)

ka : 132 : From Monkey, Wambaugh (/h)

$INIT @annotated // Compartment Declaration

AGut : 0 : Gut

CPrim : 0 : Plasma

CDeep : 0 : Deep

Cfil : 0 : Filtrate

$MAIN

double Vc = VCC * BW; // L

double Vt = k12 / k21 * Vc; // L

double Qd = k12 * Vc; // L/h

double Vfil = VfilC * BW; // L

double Tm = Tmc * BW; // mg/h

double Qfil = QfilC * QCC * pow(BW,0.74); // L/h

F_AGut = bioAv; //Example of this syntax: https://mrgsolve.org/docs/reference/house.html

////////////////////////////////////////////////////////////////////////////////

$ODE

dxdt_AGut = -ka*AGut ; // mg/h

dxdt_CPrim = 1/Vc * (ka*AGut - Qd*Free*CPrim - Qfil*CPrim*Free + Tm*Cfil/(Kt+Cfil) + Qd*CDeep);

dxdt_CDeep = 1/Vt * (Qd*Free*CPrim - Qd*CDeep);

dxdt_Cfil = 1/Vfil * (Qfil*CPrim*Free - Tm*Cfil/(Kt+Cfil) - Qfil*Cfil);

$TABLE

capture Plasma = CPrim;

capture ConcFil = Cfil;
